# SATB2 is Rarely Expressed in Endometrial or Endocervical Carcinoma

**DOI:** 10.1111/apm.70110

**Published:** 2025-12-01

**Authors:** Tatiana Amaamri‐Seebach, Mousa Mobarki, Shaqraa Musawi, Michel Péoc'h, Georgia Karpathiou

**Affiliations:** ^1^ Pathology Department University Hospital of Saint‐Etienne Saint‐Etienne France; ^2^ Department of Basic Medical Sciences (Pathology), Faculty of Medicine Jazan University Jazan Saudi Arabia; ^3^ Department of Medical Laboratory Technology, College of Nursing and Helath Sciences Jazan University Jazan Saudi Arabia

**Keywords:** adenomyoma, cervical cancer, endometrium, uterine cancer, uterus

## Abstract

Special AT‐rich sequence‐binding protein 2 (SATB2) can distinguish primary ovarian mucinous tumors from ovarian metastases of colorectal or appendiceal tumors. However, its expression in other gynecological localizations remains underexplored. Whole‐tumor sections of 376 endometrial and 27 endocervical carcinomas were examined for SATB2 expression. Among endometrial carcinomas, we found 10 cases (2.7%) expressing SATB2, all of which were endometrioid adenocarcinomas. In 8 of them, expression concerned only the morules. Only two cases expressed SATB2 in the glandular component, indicating 0.53% positivity in this cancer group. No stromal expression was detected. One case (3.8%) of endocervical adenocarcinoma focally expressed SATB2. SATB2 expression can be reliably used in the differential diagnosis of primary endometrial endometrioid and serous carcinomas as well as primary endocervical adenocarcinomas because nearly all of these tumors are SATB2‐negative. SATB2 positivity in endometrial carcinomas is almost always restricted to morules within endometrioid adenocarcinomas.

## 1 Introduction

Special AT‐rich sequence‐binding protein 2 (SATB2) has garnered interest in gynecological pathology because of its utility in distinguishing primary ovarian mucinous tumors from ovarian metastases of colorectal or appendiceal tumors [1]. SATB2 is a transcriptional regulator that plays an important role in osteoblastic differentiation; it is positive in most benign and malignant tumors with osteoblastic differentiation, such as osteosarcomas, osteoblastomas, osteoid osteomas, and fibrous dysplasias [2]. Beyond this role in bone physiology and pathophysiology, it is expressed in most colorectal carcinomas [3], raising the question of whether this marker could aid in the challenging differential diagnosis of ovarian mucinous neoplasms and ovarian endometrioid tumors, both of which may mimic metastatic colorectal adenocarcinoma [1]. Moh et al. conducted a tissue microarray study of primary ovarian mucinous tumors (34 cystadenomas, 60 borderline tumors, and 17 adenocarcinomas), 72 ovarian endometrioid adenocarcinomas, 3 ovarian endometrioid borderline tumors, 69 ovarian metastatic gastrointestinal tumors, and 4 ovarian metastatic endocervical carcinomas [1]. None of the ovarian endometrioid tumors or primary ovarian mucinous carcinomas expressed SATB2 [1]. One borderline mucinous tumor and four benign mucinous tumors (all associated with teratomas) expressed SATB2 [1]. In contrast, 75% of the metastatic colorectal and 86% of the metastatic appendiceal tumors expressed this marker [1]. SATB2 was not expressed in any of the metastatic pancreatic, gallbladder, gastric, or endocervical adenocarcinomas [1].

These findings clarified the diagnostic utility of SATB2 in ovarian mucinous and endometrioid carcinomas. However, data regarding SATB2 for the endometrial or endocervical primaries are largely lacking. Thus, we aimed to investigate SATB2 expression in a series of uterine adenocarcinomas to ensure that this marker can be reliably considered in the differential diagnosis of these primaries when a colorectal origin is suspected.

## 2 Material and Methods

This retrospective, non‐interventional study was performed in the Pathology Department of the University Hospital of Saint‐Etienne, France, and approved by the local ethics committee (IRBN1172024/CHUSTE). The study included consecutive hysterectomy specimens of endometrial cancer (endometrioid adenocarcinoma or serous carcinoma) and consecutive cases of usual‐type, HPV‐associated endocervical adenocarcinoma (biopsy or excision specimens), classified according to previously suggested criteria [4]. Other histological types were excluded because of the small number of cases.

Formalin‐fixed paraffin‐embedded 4‐μm‐thick whole‐tumor sections were used for immunohistochemistry in an automated staining system (OMNIS, Dako‐Agilent, Santa Clara, CA, USA) with SATB2 (clone EP281, rabbit monoclonal, 1/100 BioSB). Nuclear expression in the tumor or nearby normal cells was recorded.

## 3 Results

**FIGURE 1:**
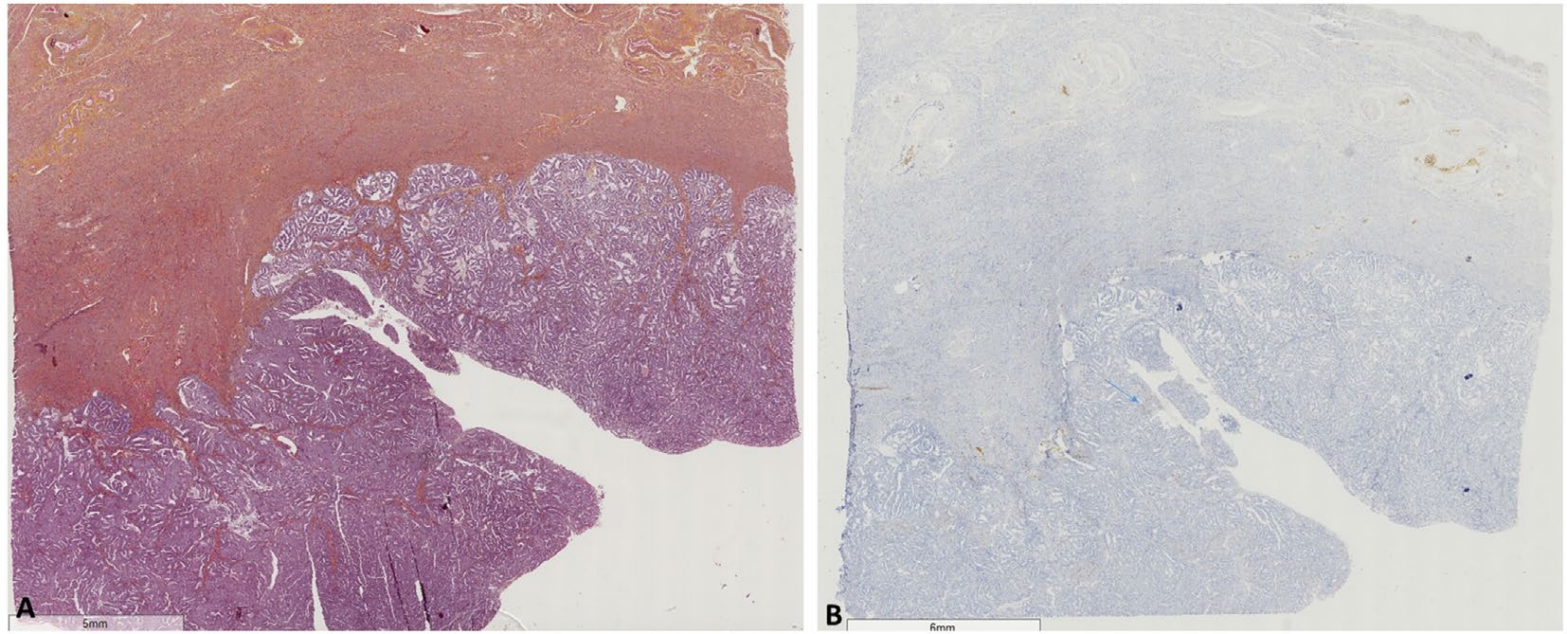
Endometrioid adenocarcinoma (A. Hematoxylin, eosin, safran ×4) showing only focal (arrow) SATB2 expression (B. ×4).

**FIGURE 2:**
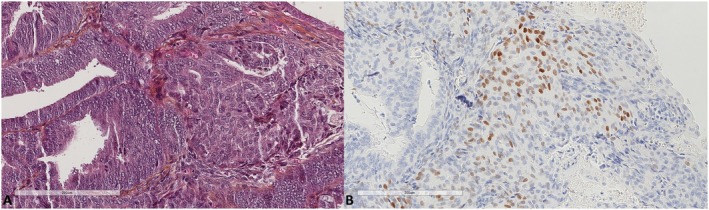
The same case as in Figure 1 in higher magnification, showing that the positive focus corresponded to morule formation (A. Hematoxylin, eosin, safran ×200, B. SATB2 ×200).

**FIGURE 3:**
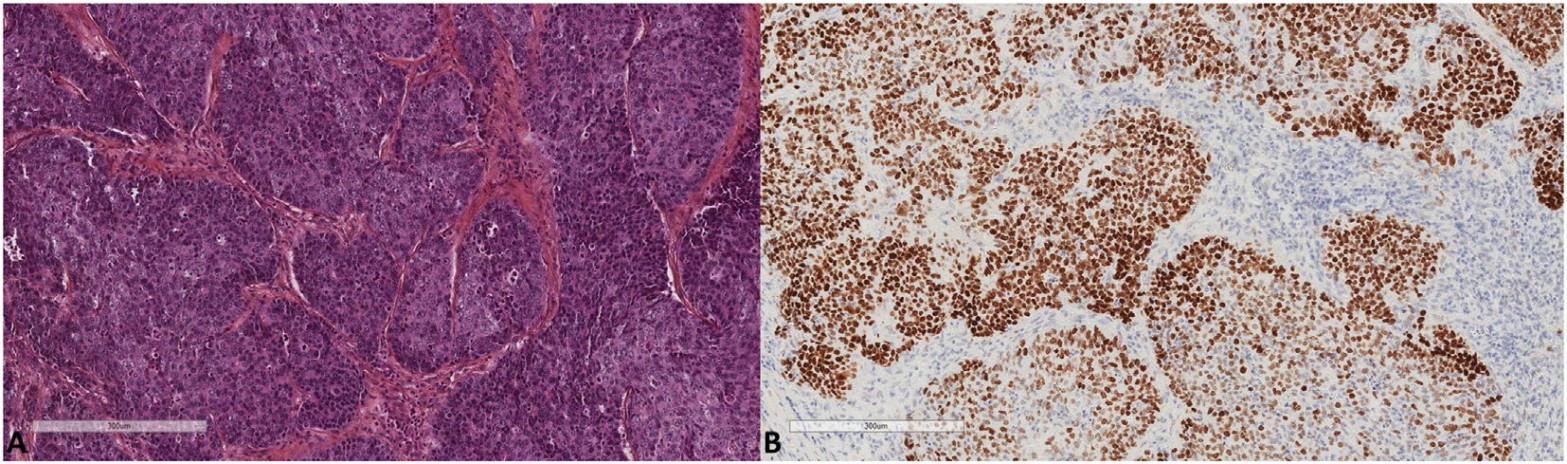
Grade 3 endometrioid adenocarcinoma with diffuse SATB2 expression (A. Hematoxylin, eosin, safran ×100, B. SATB2 ×100).

**FIGURE 4:**
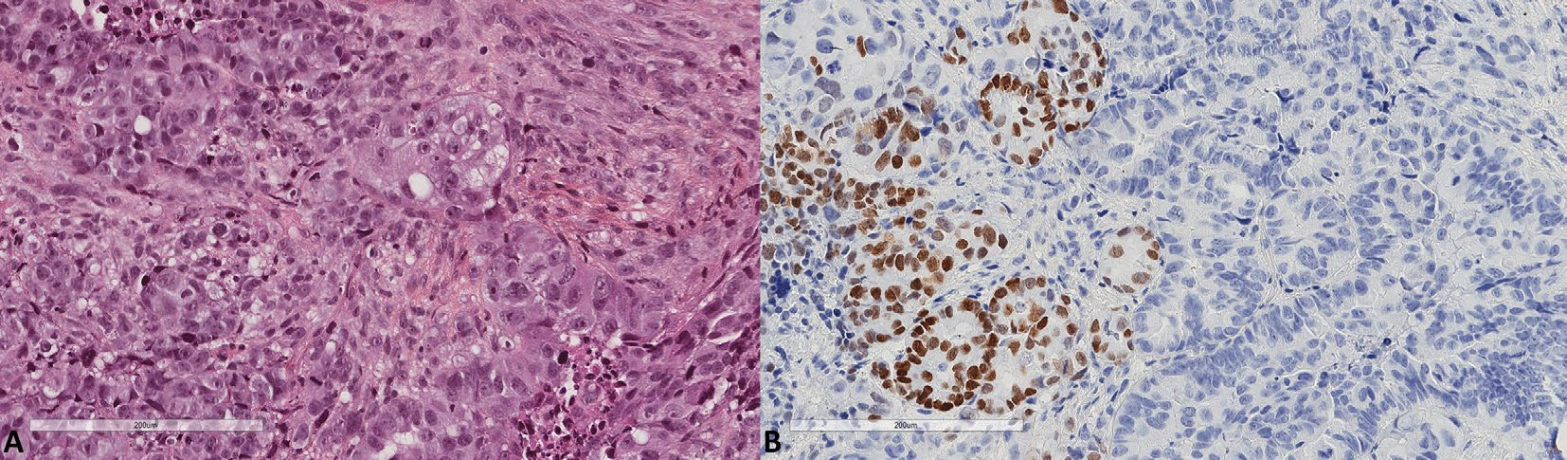
Endocervical adenocarcinoma with focal SATB2 expression (A. Hematoxylin, eosin, safran ×200, B. SATB2 ×200).

We studied 376 full tumor sections of hysterectomy specimens from 320 and 56 patients treated for primary uterine endometrioid adenocarcinoma and primary uterine serous carcinoma, respectively. Endometrioid adenocarcinoma was classified as grade 1 (*n* = 130, 40.6%), grade 2 (*n* = 150, 46.9%), or grade 3 (*n* = 40, 12.5%). Age at diagnosis ranged from 33 to 93 years (69.2 ± 11.1). We found 10 cases (2.7%) expressing SATB2, all of which were endometrioid adenocarcinomas. In eight of them, the expression was confined to morules (Figures 1 and 2). Only two cases expressed SATB2 in the glandular component (Figure 3), indicating 0.53% positivity in the endometrial cancer group. Endocervical adenocarcinomas (*n* = 27) were diagnosed in women whose age ranged from 35 to 77 years (57.6 ± 13.5). One case (3.8%) showed very focal expression of SATB2 (1% of tumor cells, Figure 4).

## 4 Discussion

Previous studies on the expression of SATB2 in the uterus have focused on endocervical adenocarcinomas, endometrial polypoid adenomyomas, uterine sarcomas, and carcinosarcomas. In a multicenter study, Stolnicu et al. used 297 endocervical adenocarcinomas to develop tissue microarrays and perform a broad immunohistochemical panel, including SATB2 with the same EP281 clone [5]. They identified one usual‐type endocervical adenocarcinoma (0.7%), one endocervical clear cell carcinoma (14.3%), and one endocervical endometroid adenocarcinoma (33.3%) expressing SATB2 [5]. Our study detected one usual‐type endocervical adenocarcinoma (3.8%) focally expressing SATB2. The slightly higher percentage in our study can be explained by the fact that Stolnicu et al. used tissue microarrays, which could miss focal staining; furthermore, they used a 5% cutoff value for SATB2, so a more focal staining—such as the 1% found herein—would not have been detected [5]. Using another SATB2 clone (EPNCIR130A) and a cutoff point of 10% positive tumor cells, Giannico et al. [6] showed expression in one (4%) of 26 endocervical adenocarcinomas (type not described), similar to our study. Our results confirm that SATB2 expression in usual‐type endocervical adenocarcinoma is rare and focal; thus, it can be reliably used to exclude a colorectal origin. Interestingly, even the cases with mucinous, gastric, or intestinal differentiation did not show SATB2 expression in Stolnicu et al.'s study [5], further highlighting the utility of this marker.

SATB2 has been proposed as a useful diagnostic marker for atypical polypoid adenomyoma, another uterine entity. McCluggage and Van de Vijner were the first to show that SATB2 is consistently expressed within morules in various endometrioid lesions as well as by the stromal cells of the atypical polypoid adenomyoma [7]. The authors examined 43 morules and 13 nonmorular squamous metaplasias in various endometrial and ovarian lesions, as well as various endometrioid polypoid lesions. The cases with morules corresponded to low‐grade endometrial and ovarian endometrioid adenocarcinomas, ovarian borderline endometroid tumors, endometrial hyperplasias, and atypical polypoid adenomyomas (6 cases). Nonmorular squamous metaplasia comprised low‐grade endometrial and ovarian endometrioid adenocarcinomas. In addition to atypical polypoid adenomyomas, the endometrial polypoid lesions included 12 endometrial polyps, 11 uterine adenosarcomas, and 5 adenomyomatous polyps. Immunohistochemical analysis was performed in two laboratories with different immunostainers and antibodies (one polyclonal and one monoclonal—clone not described). Of the 43 morules, 38 were positive, whereas 13 squamous metaplasias were negative. The stroma of the six atypical polypoid adenomyomas was positive, whereas occasional stromal staining was observed in one endometrial polyp and four adenosarcomas. The reason for this expression in the morules or the stroma is unknown [7]. Following this observation, Worrell et al. examined 32 atypical polypoid adenomyomas, 39 adenomyomatous polyps, and 30 endometrial carcinomas, sampled over a 31‐year period, to determine whether stromal SATB2 expression (the same EP281 clone as in the current study but performed manually) could be useful in their distinction [8]. They reported stromal expression in 94% of atypical polypoid adenomyomas, none of the polyps, and 17% of adenocarcinomas; notably, some SATB2 expression was detected in normal structures, such as endometrial stroma, myometrium, and vessels [8], although we did not find any such nonspecific expression. Morules were frequently positive (almost 80% of 44 cases) regardless of lesion type [8]. In our large cohort, we confirmed that there is no stromal SATB2 expression; thus, this expression can be used to distinguish an atypical polypoid adenomyoma from an endometrial carcinoma. In addition to morules, we showed that SATB2 expression by tumor cells is extremely rare in endometrial cancer; thus, SATB2 can be reliably used in the diagnostic evaluation of a uterine endometroid or serous carcinoma, if necessary. This contrasts with the results of another study [9], which found SATB2 expression in 8/19 (42%) uterine carcinosarcomas (inside the sarcomatous portion), 10/26 (38%) grade 3 endometrial endometrioid carcinomas, 11/30 (37%) undifferentiated/dedifferentiated endometrial carcinomas, and 6/22 (27%) uterine serous carcinomas, while none of the 15 clear cell carcinomas were SATB2 positive. Tumors were sampled over a 16‐year period, and whole‐tumor sections with a cutoff value of 5% positivity in tumor cells were examined for the expression of the same EP281 clone. These results therefore contradict our results regarding uterine endometrioid and serous carcinomas as well as previous results related to ovarian endometrioid tumors [1]. The authors do not clarify whether they considered morule expression in the reported positivity; the eventual stromal positivity is also not reported. They reported a range of tumor cell expression between 5% and 90%, with the median values varying from 5% to 20%; the median tumor cell expression for grade 3 endometrioid adenocarcinomas and serous carcinomas was 5%. Notably, SATB2 is expressed in 49% of primary adenocarcinomas of the urinary bladder and 20% of urothelial carcinomas with glandular differentiation [6].

Regarding uterine mesenchymal tumors, SATB2 expression has been studied in 71 whole‐tumor sections of variable lesions comprising endometrial stromal nodules, low‐grade endometrial stromal sarcomas, leiomyomas, leiomyosarcomas, undifferentiated sarcomas, adenosarcomas, and carcinosarcomas—and in tissue microarrays of 78 more tumors—using a cutoff value of 10% [10]. SATB2 was positive in 83% of low‐grade endometrial stromal sarcomas, 40% of undifferentiated sarcomas, 13% of leiomyosarcomas, 14% of adenosarcomas, and 8% of carcinosarcomas [10]. Another study of 60 gynecological carcinosarcomas showed SATB2 expression in the foci of osteosarcoma; however, it was also positive in 60% of cases lacking osteosarcoma, mostly in the undifferentiated foci [11].

In conclusion, we showed that SATB2 expression is extremely rare in a large series of primary endometrial endometroid and serous carcinomas as well as in a small series of endocervical adenocarcinomas. Stromal SATB2 expression was also absent. Thus, this marker can be reliably used in the differential diagnosis of primary uterine adenocarcinomas from other primaries.

## Funding

The authors received no specific funding for this work.

## Conflicts of Interest

The authors declare no conflicts of interest.

## Data Availability

The data that support the findings of this study are available from the corresponding author upon reasonable request.
